# Admission Shock Index in Acute Aortic Dissection: A Triage Trigger or a Restated Vital Sign?

**DOI:** 10.1002/clc.70368

**Published:** 2026-06-06

**Authors:** Shaher Yar, Youmna Imtiaz, Haniyah Attiq, Ishvah Shashwat Nag

**Affiliations:** ^1^ The Wright Centre for Graduate Medical Education Scranton Pennsylvania USA; ^2^ University College of Medicine and Dentistry Lahore Pakistan; ^3^ Danylo Halytsky Lviv National Medical University Lviv Ukraine

We read with interest the report by He et al. evaluating admission shock index (SI) as a predictor of in‐hospital all‐cause mortality in 1250 patients with acute aortic dissection (AD) and intramural hematoma (IMH) [1]. The authors deserve credit for a sizeable 6‐year cohort, restricted cubic spline analysis, and external validation in the eICU database, rarely seen in retrospective work. We share their interest in pragmatic bedside triage tools and offer the following observations.

First, the diagnostic performance of the SI threshold of 0.6 is modest. The AUC of 0.62 in the primary cohort and 0.63 in the eICU cohort sits only marginally above chance, and the Youden‐derived cut‐point yields a sensitivity of 0.46 and a specificity of 0.76. A rule that misses more than half of patients destined to die in the hospital is unlikely to function as the “early warning trigger” the discussion envisages. The Liu cut‐point raises sensitivity to 0.53 at lower specificity. These figures deserve more prominence in the abstract, since the paper's central clinical claim rests on them.

Second, the prognostic independence of SI is not fully demonstrated. The fully adjusted Cox model accounts for a history of hypertension but does not adjust for admission systolic blood pressure or heart rate as continuous variables, the two components from which SI is mechanically derived. Bossone et al., cited by the authors, established a J‐curve between presenting SBP and in‐hospital mortality in 6238 IRAD patients [2], and the authors also cite work identifying heart rate as independently prognostic. A nested model comparing SI against SBP alone, HR alone, and both as separate predictors would clarify whether SI adds discrimination or restates information already carried by its constituents.

Third, the interaction between SI and treatment strategy is striking but susceptible to confounding by indication and immortal time bias [3]. Patients who die early have no opportunity to receive surgery or TEVAR, and surgical candidacy itself depends partly on hemodynamic stability and Stanford classification. Treating intervention as a baseline variable rather than a time‐varying exposure tends to inflate the apparent benefit of surgery in those who survive long enough to receive it. A landmark or time‐varying analysis would address this.

A smaller note: the restricted cubic spline shows a linear association in all three cohorts (P‐nonlinear consistently above 0.28). When the underlying relationship is linear, dichotomizing at 0.6 discards information and risks residual confounding and spurious threshold effects [4]. Modeling SI continuously would be more efficient and avoid implying a biologically distinct cut‐point where the data do not support one.

None of this detracts from the importance of the question. We would suggest a prospective cohort with prespecified vital‐sign thresholds, adjustment for SBP and HR as continuous covariates, time‐varying treatment exposure, and decision‐curve or net‐reclassification comparison against simpler markers. Confirming the incremental value of SI under tighter conditions would strengthen its claim to a place in routine triage.

We thank the authors for raising an important question and the editors for the opportunity to comment.

Yours sincerely,

The authors

## Funding

The authors have nothing to report.

## Ethics Statement

As this is a commentary on a published study and no new data were collected or analyzed, ethics approval was not required.

## Conflicts of Interest

The authors declare no conflicts of interest.

## Data Availability

Data sharing is not applicable as no new data was generated, or the article describes entirely theoretical research.

## References

[1] L. He , Y. Chen , C. Tian , et al., “Admission Shock Index Is an Independent Predictor of In‐Hospital All‐Cause Mortality in Patients With Acute Aortic Dissection and Intramural Hematoma,” Clinical Cardiology 49, no. 5 (2026): e70333, 10.1002/clc.70333. 42041137 PMC13112594

[2] E. Bossone , R. Gorla , T. M. LaBounty , et al., “Presenting Systolic Blood Pressure and Outcomes in Patients With Acute Aortic Dissection,” Journal of the American College of Cardiology 71, no. 13 (2018): 1432–1440, 10.1016/j.jacc.2018.01.064. 29598863

[3] S. Suissa , “Immortal Time Bias in Pharmacoepidemiology,” American Journal of Epidemiology 167, no. 4 (2008): 492–499, 10.1093/aje/kwm324. 18056625

[4] P. Royston , D. G. Altman , and W. Sauerbrei , “Dichotomizing Continuous Predictors in Multiple Regression: A Bad Idea,” Statistics in Medicine 25, no. 1 (2006): 127–141, 10.1002/sim.2331. 16217841

